# Methods for Generating Year-Round Access to Amphioxus in the Laboratory

**DOI:** 10.1371/journal.pone.0071599

**Published:** 2013-08-26

**Authors:** Èlia Benito-Gutiérrez, Hermann Weber, Diana Virginia Bryant, Detlev Arendt

**Affiliations:** Developmental Biology Unit, European Molecular Biology Laboratory (EMBL), Heidelberg, Germany; Laboratoire Arago, France

## Abstract

Cephalochordates, commonly known as amphioxus, are key to understanding vertebrate origins. However, laboratory work suffers from limited access to adults and embryonic material. Here we report the design and experimental validation of an inland marine facility that allows establishing stable amphioxus colonies in the laboratory and obtaining embryos at any time of day and over almost the entire year, far exceeding natural conditions. This is achieved by mimicking the natural benthic environment, natural day- and moon- light, natural substrate and by providing a strictly controlled and seasonally fluctuating temperature regimen. Moreover, supplemented algae diets allow animals to refill their gonads in consecutive years. Spontaneous spawning, a major problem in previous setups, no longer occurs in our facility; instead, all breeding is induced and fertilization occurs fully *in vitro*. Our system makes amphioxus a standard laboratory animal model.

## Introduction

The past decades have seen renewed interest in amphioxus as a model system that sheds light on the invertebrate-vertebrate transition and the molecular basis for the vertebrate radiation [1]–[3]. However, the use of amphioxus in molecular genetics and developmental biology has been hampered by technical limitations such as limited access to embryonic material, precluding experimentation on a daily basis. Consequently, current studies have remained largely descriptive (with a few exceptions [4]–[7].

To overcome these limitations, various approaches have been developed to facilitate access to amphioxus in the lab [8]–[14]. However, these studies disagree on the husbandry conditions in an artificial marine environment. Moreover, all systems previously described suffer from recurrent spontaneous spawning in the parental tanks, thus limiting the breeding period in captivity to only a couple of months of each year or just sporadically [9]–[12], [14]–[16]. Since spontaneous spawning in these systems are semi-synchronous and in line with the natural breeding season (e.g. [14]), animals appear to still follow the natural cycle.

Here, we describe a new facility and robust protocols for long-term husbandry and efficient and controlled breeding of different amphioxus (species, genus or populations) of interest, including: a powerful refrigeration system that reproduces a broad range of temperatures from 7°C to 27°C, which covers virtually all naturally occurring temperature ranges in world-wide habitats; the ability to mimic any natural photoperiod, moonlight intensity and twilight (dusk and dawn); a self-purifying flow-through system, which allows efficient washout of organic matter and other contaminants.

We report the establishment of stable colonies for various populations of the European species, *Branchiostoma lanceolatum*. All populations show substantially improved long-term survival rates, with values above 95% survival in more than 2-years-long cultures, in contrast to the 83.3% survival previously achieved in 1 year-long cultures (with no survival reported for subsequent years) [9]–[14]. We obtain strongly expanded annual breeding seasons of up to nine months, in contrast to the two-month-breeding season previously achieved in *B.lanceolatum* [9]–[19]. This represents a 4.5-fold expansion respect from any other system previously reported and, in addition, allows controlling the beginning and the end of the expanded breeding season thus decoupling the animals from their natural cycle. Providing special dietary supplements, we observe gonad refill in line with the seasonal fluctuations programmed in the facility and animals spawning in consecutive years post collection. Furthermore, and in contrast to previous systems, the animals in our facility spawn after induction only. By manipulating the temperature and light conditions, spawning can be programmed at desired times and days according to the experimental needs.

Our facility dramatically improves the conditions of working with amphioxus and thus facilitates efforts to make it a model species fully accessible to *in vivo* experimental manipulation, imaging, stock keeping and any approach that requires vast amounts of animal material.

## Materials and Methods

### Ethics Statement

This research did not involve the use of any vertebrate animal. This research was carried on non-protected and non-endangered invertebrates only. No animals were sacrificed for the work reported here.

*Branchiostoma lanceolatum* specimens were collected in Helgoland (Germany), Roscoff or Argelès-sur-mer (France) by dredging. All animal collections were performed according to the recommendations and regulations in the respective Marine Stations: AWI, Station Biologique Roscoff and Observatoire Oceanologique de Banyuls-sur-mer. Research was done under the supervision of the responsible bodies in the respective Marine Stations and under the framework of the Association of European Marine Biological Laboratories (ASSEMBLE) approved by the European Union (FP7).

### Facility design and husbandry conditions

**Figure 1:**
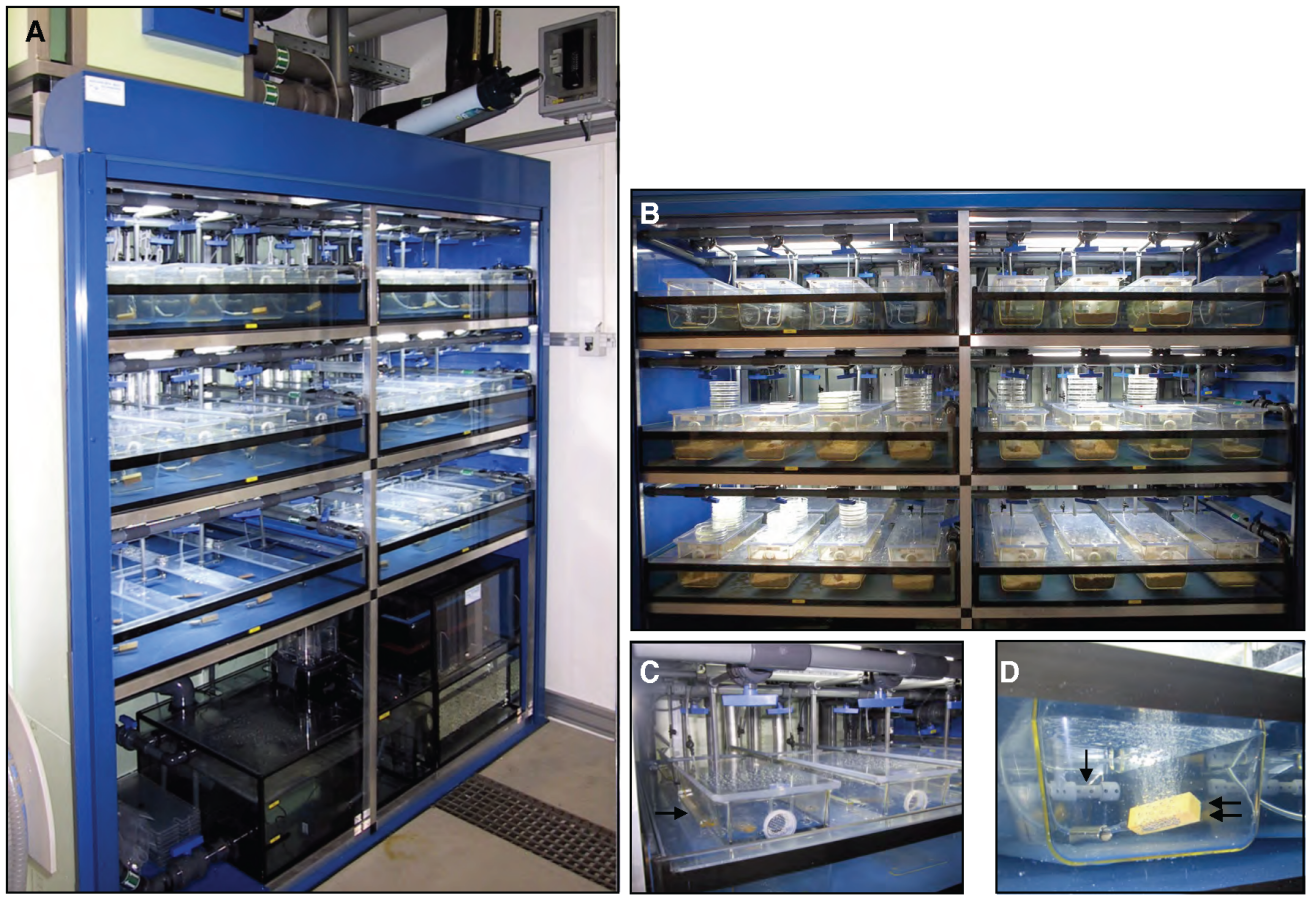
The amphioxus facility. (**A**) Lightproof cabinet of the facility, with open shutters, showing three shelves with two main tanks each. The upper distribution tank and UV sterilisation device are visible at the top outside the cabinet. The two-compartment lower reservoir tank and neighbouring filter unit are at the base of the facility inside the cabinet. (**B**) View of the six main tanks encapsulating eight amphioxus boxes each, distributed in two rows of four. All tanks in use are sand-bedded. (**C**) Encapsulated tank system: the amphioxus boxes are encapsulated into the main tank through a fitted PVC grid (arrow), leaving two thirds of their height immersed in the main tank. The unique unsealed part is the netted outlet (double arrow) of the amphioxus boxes, used for the flow-through circulation of the seawater. (**D**) All amphioxus boxes are equipped with inverted T-shape water jets, multi-perforated at the base (arrow). This was conceived to generate a unidirectional seawater wave washing out the entire width of the boxes. In addition, all amphioxus boxes are equipped with 5 cm air stones (double arrow) to constantly oxygenate the water by dispersion.

In order to simulate the benthic marine ecosystem where wild amphioxus live, we engineered an automated marine facility with a closed circuit of constantly flowing-through natural sea water (Figure S2), with natural daylight, dusk and dawn, natural moon phases and sand-bedding (Fig. 1 and Figure S1). For a technical description of the basic framing, the encapsulated tank system, the closed water circuitry, light instalments, oxygen supply and water quality control see Materials and Methods S1.

Animals were cultured at a density of 25–30 animals per box. Annual temperature curves followed records of the SOLA station (SOMLIT Observatory Laboratoire Arago) close to the Argelès sampling site (http://somlit-db.epoc.u-bordeaux1.fr) (see Table S1). In all cases, pH and salinity values stabilized around pH 7.9–8.1 and 54–56 ms. Animals are fed on seven algal species grown in a custom-made algae facility (Figure S4).

### Survival and Spawning Data

Routine inventories were carried out every six months and survivor numbers were determined by subtracting the number of dead animals from the number of inventoried animals. Adaptation was calculated on the number of dead animals in the first month subtracted from the number of animals collected. Spawning efficiency is defined as the percentage of spawning animals among the total subjected to heat shock. The raw data for survival and spawning records are displayed in Table S2 and Figure S5. Tables and graphs were done in ‘Numbers’ (iWork, Apple) and statistical analyses in R (The R foundation for Statistical Computing).

## Results and Discussion

### Stable amphioxus colonies in the laboratory

**Figure 2:**
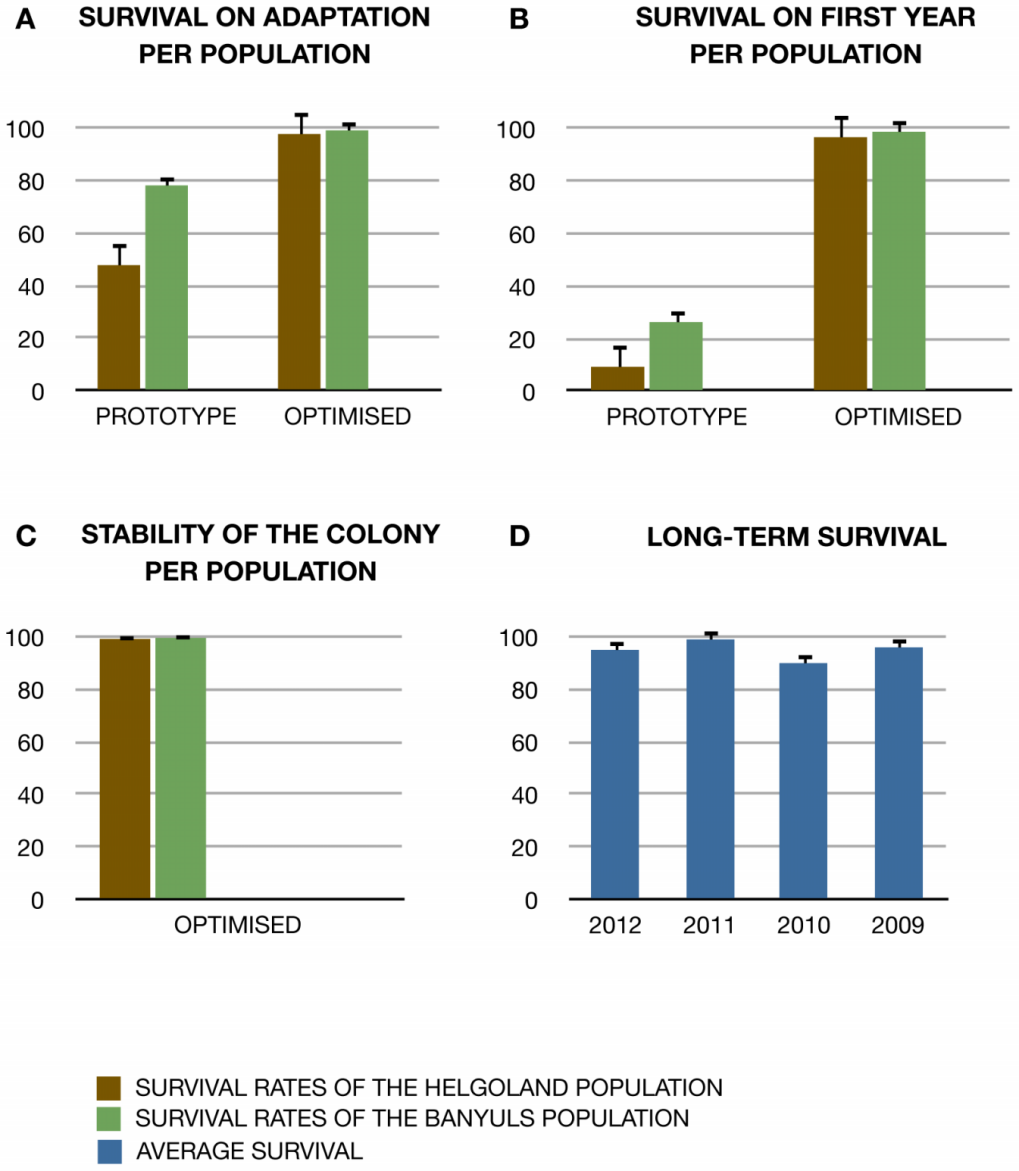
Long-term survival and stability of amphioxus colonies. (**A**) Survival rates during the first month of farming in the laboratory per population in “prototype” and “optimized” setups. (**B**) Survival rates during the first year of farming in the laboratory per population in the two culturing setups. (**C**) Survival of the different amphioxus populations in our optimised facility over 4 years of farming. (**D**) Average survival rates in 2012 of animals that were farmed for several years, meaning they were collected in 2012, 2011, 2010 or 2009. Confidence intervals on the binomial probabilities were generated using the R package “binom” with the exact method.

The major cause of mortality for cultured amphioxus is bacterial infection, a recurring problem in all previous attempts to culture amphioxus for longer periods [13]. To overcome this limitation, our approach mimics natural conditions as closely as possible (see Materials and Methods and Materials and Methods S1). Whilst most quantified parameters (e.g., salinity, pH, day and night ratios and twilight times) were kept constant throughout the different conditions assayed, we varied culturing conditions (“prototype” and “optimized” facility; see materials and methods S1 and table S1) in two important points that proved to significantly improve our survival rates from an average of 20% to an average of 97.8% of survival in our cultures (see Fig. 2B): (1) Commercial sterile sand bedding was replaced with natural sand collected from the original habitats in the optimised facility; and (2) Natural annual temperature curves were lowered by three degrees in average in the optimised facility. Data on the performance of both facilities was collected (see raw data in Table S2) and further compared with regard to adaptation of newly collected animals, long term maintenance and resistance to fluctuating temperatures (important for controlled breeding, see below).

#### Improved Adaptation

Upon arrival all animals are closely monitored (see Protocol S1). As a measure of successful adaptation to the facility we calculated the survival rates during this period. While some populations consistently adapted better than others, in the optimized facility the population differences were reduced, resulting in overall improved survival rates (around 92%), independently of the site of origin (p = 0.005; Fig. 2A). In order to exploit a good-sized starting population this first month of farming is critical. In this regard, care should be emphasized during the collection and transportation of the animals, which might affect the health status of the animals and their capabilities to adapt to the new environment (See Protocol S1).

#### Long-term maintenance

While satisfactory short-term survival rates had been previously observed using other culturing systems, these usually decline dramatically over time [11], [14]. In this regard, the most distinguishing feature of the optimized facility is that long-term survival rates stabilise at an even higher value than during adaptation, with an observed average survival of ∼95% during the first year in the optimised facility (Fig. 2B). By contrast, in the prototype facility, first year-survival depended on population origin. Moreover, the survival rates in the optimised facility further improved in subsequent years, stabilising at a value close to 100% (Fig. 2C). Thus, the optimised facility enables the establishment of stable colonies of amphioxus with overall survival rates above 95% (Fig. 2D).

#### Resistance to fluctuating temperatures

Importantly, survival rates in the optimised facility were robust to fluctuations in the temperature at which the animals were cultured. This is important since such fluctuations typically arise in nature and are required for controlled maturation of the gonads and subsequent breeding (see below).

### Gonad refill in captivity

**Figure 3:**
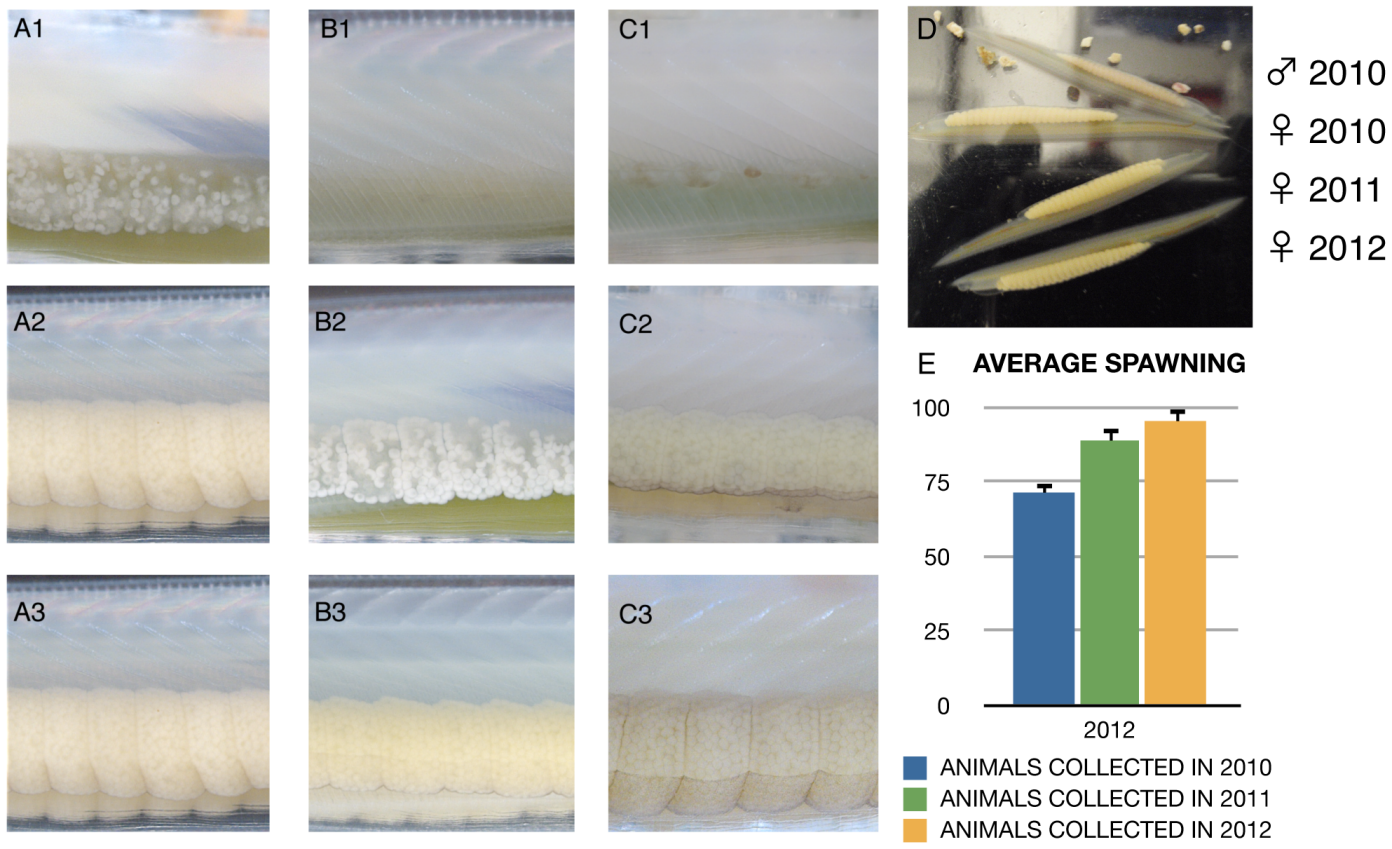
Annual gonad refill in the amphioxus facility. (**A–C**) Gonad status of maturation for three different groups of animals in our optimised amphioxus facility: (**A**) Animals collected with incipient gonads; (**B**) Animals collected with no gonads or animals that spawned in the facility in previous years; (**C**) Animals already spawned in the same breeding season.1 is the initial status of the gonads for a given individual, and its progression (2) until the gonads are fully developed (3). State 3 is achieved in our facility in line with artificial seasonal fluctuations only in combination with feeding on Food Mix C. In state 3 most of our animals are able to spawn after being induced. (**D**) Comparison between animals that refilled their gonads in the facility or in the wild. The picture shows a snapshot of ripe animals in our facility in 2012, with animals collected in 2010 and 2011 versus a female collected in 2012 (with refilled gonads in the wild). By feeding on Food Mix C, no appreciable difference is seen between animals that maturated their gonads in the nature or in the facility. (**E**) Annual spawning percentages of animals collected in 2010 and 2011 that spawned in subsequent years. The spawning in subsequent years appears to improve over the time with a maximum difference of a 30% between 2010 and 2012 animals that spawned in 2012.

Unsurprisingly, previous studies have identified proper feeding as playing a critical role in the refill of gonads of cultured amphioxus, (albeit, such refilling was restricted to the natural spawning season [9]–[10]. Beyond this, little is known about external cues and hormonal signals that induce gonad development and maturation in amphioxus and all efforts to boost gametogenesis have failed thus far [11]. As we initially observed stasis in the gonadal sacs of long-term surviving animals that had been fed on microalgae only, we tested different kinds of diets with increased variety of both micro- and macro-algae, cultured in situ (See Figure S4), alongside various supplements containing vitamins, essential amino acids, protein, fatty acids and iodine. Three different food mixes were tested; A) fresh algae mix, as in previous culturing systems; B) fresh algae supplemented with vitamins and essential amino acids; and C) as in B but also enriched in lipids, protein and iodine. This was fed into different tanks containing animals without gonads, with incipient gonads or with no gonads at all, either after collection or after spawning in the facility (independently of year), so we could observe the general status of gonad maturation per tank (see Fig. 3 for more information about the animal groups tested).

While, as previously observed [9]–[10] food mix A allowed completion of gonad maturation in animals freshly collected during the natural maturation phase (Fig. 3A1–A3), it was insufficient to trigger gametogenesis in animals that, at the time of collection, had not started gonad maturation (Fig. 3B1). This means that food mix A is only effective in about a 70% of the collected animals, which is the average percentage of animals with gonads in our annual collections (for dates see Table S2). By contrast, food Mix B enabled sustained gonad maturation in animals without visible gonads at the time of collection, meaning that mix B is sufficient to prepare an entire batch of freshly collected animals for spawning, independently of their gonad status at the time of collection. However, it did not allow gonad maturation in subsequent years (Fig. 3B1–B3). Only with food Mix C could we observe gonad maturation in captivity in the breeding seasons of subsequent years. (Fig. 3B and 3C). Food Mix C sustained gonad refill of both animals that had spawned in the natural breeding season in successive years and animals that had spawned outside of the natural breeding season. Gonad refill in captivity appears to occur as in nature [17] (Fig. 3A–D), but is obtained in full accordance with the artificial seasonal light and temperature regimen in the facility, which demonstrates that the animals are uncoupled from the natural cycle and fully adopted to the artificial environment. Once food mix C was in place, animals collected in 2010 and 2011 spawned controllably in subsequent years (Fig. 3 E). Among the animals in which spawning was induced, the success rate of spawning was high (62.5% and 71.42% success rate for the 2010 animals in 2011 and 2012; 88.88% for the 2011 animals spawned in 2012). Still, our numbers are small with only a 8% of our long-term cultured population ready to spawn at any time of the year, meaning that further improvement in diet is necessary to get independent from annual collections. Consecutive spawnings have been recently reported for *B. belcheri* [18] but in contrast to our fully controlled system these occur spontaneously in the parental tanks. Therefore, our facility is the first to allow controlled consecutive spawnings in the laboratory, which is critical for exploiting amphioxus in the laboratory.

### Artificial expansion of the spawning season

**Figure 4:**
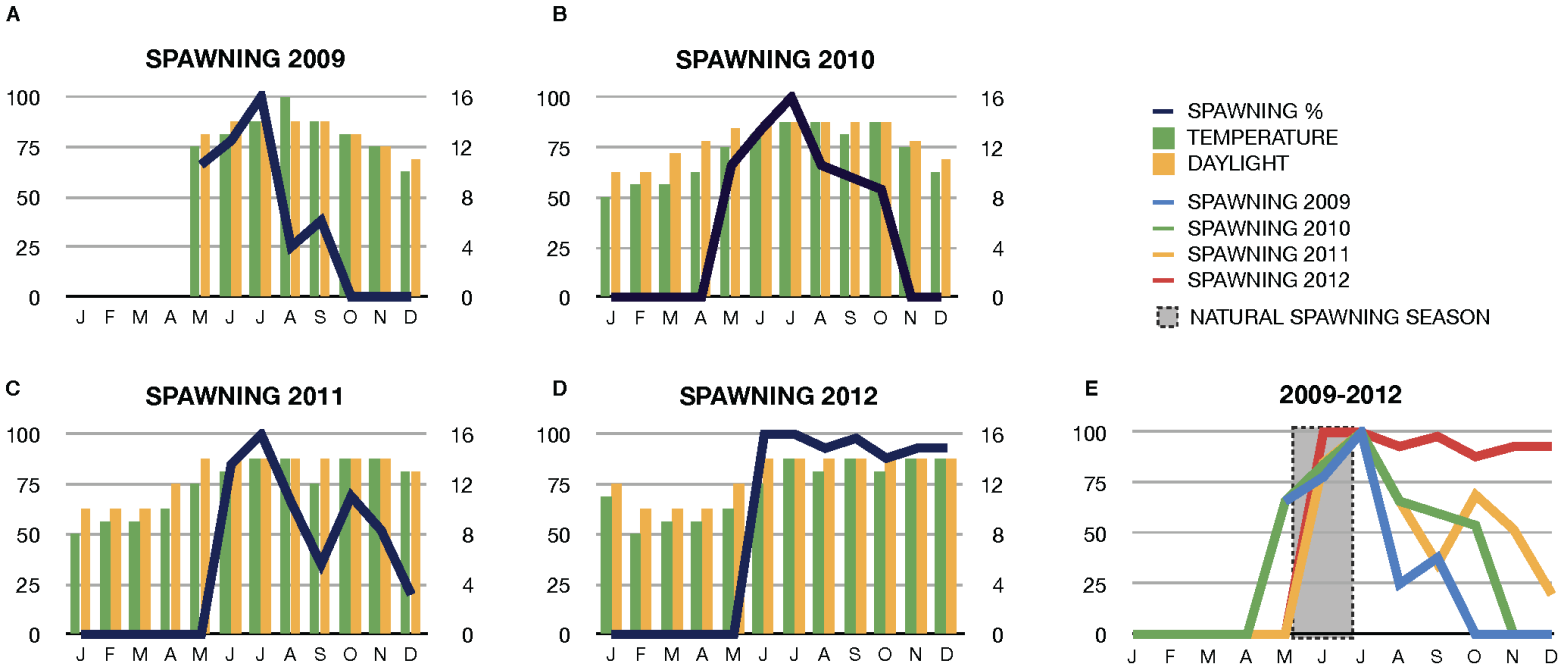
Expansion of the spawning season in the amphioxus facility. (**A–D**) Annual spawning periods and efficiencies in 2009, 2010, 2011 and 2012. The results show that the animals respond to the artificial changes, meaning deliberate fluctuations of temperature and light, which provoked a second peak of spawning, specially pronounced in 2011 (**C**) and maintaining spawning efficiencies no lower that 89% all through the facility spawning season in 2012 (**E**). Progressive expansion of the spawning season in the facility in the last four years and increment of the spawning efficiencies over the time, in-line and off-line the natural spawning season in the wild.

Until now, a major drawback in amphioxus research has been the limited time window during which embryos can be obtained (at present ∼2 months per year during the natural breeding season in *B.lanceolatum* [9]–[10], [15], [19]). As it was suggested that the raise in temperature during springtime was important for successful spawning [9] we tested the effect of different temperature regimens and daylight/moonlight ratios upon the readiness of the animals to spawn in captivity. To this aim the facility is equipped with a control panel that allows manipulating and recreating natural seasonal fluctuations (See Figure S3). We observed that animals were ready to spawn shortly after entering the artificial summer cycle and, importantly, artificially delaying the start of the autumn program prolonged the breeding season for at least an additional eight months (Fig. 4). Maximal spawning efficiency (100% of animals spawning) was reached in July, followed by a gradual decrease during subsequent months (Fig. 4). However, critically, the spawning efficiency was more than sufficient to enable the collection of embryos for experimental work until late in the year. To further increase spawning efficiency during the entire artificial season we exposed the animals to mild fluctuations in the seasonal temperature regimen. In essence, we generated short periods of colder days before raising the temperature back to standard summer values. This was motivated by the observation that this pattern arises in nature in late spring shortly before the spawning season starts [9]. This enabled a second peak in spawning efficiency to be obtained, which was especially pronounced in 2011, where a spawning efficiency of 71% was observed in October (Fig. 4C). In 2012 mild drops in the temperature allowed us to maintain the spawning efficiency at a high level throughout all eight months, with 93% efficiency in December (Fig. 4D) and 75% efficiency in January 2013 (see Figure S5). Importantly, we have successfully expanded the spawning season in each of the last four years, with increasing efficiency (Fig. 4E). In 2012 spawning efficiency was never lower than 89% and continued into January 2013 (Fig. 4D). Therefore, our artificially expanded and fluctuating seasons generate a pool of responsive animals that can be induced to spawn in a controlled manner (see below) throughout almost the entire year (Fig. 4E).

### Controlled spawning at any time of day

As wild amphioxus spawn after sunset, we attempted to control the timing of spawning by varying daylight times. In our facility, the amphioxus shed their gametes almost exactly one hour after sunset, if exposed to heat shock before (see Protocol S2 adapted from 9 and 10). We thus kept the animals in light-proof boxes during the thermal shock and delayed the onset of darkness in incremental steps. By delaying the off time of the bulb inside the light box the animals were induced to spawn at specific times (always one hour after the light was extinguished). Such programmed spawning was effective for up to 10 hours after the normal sunset time in the facility. Since *in vivo* manipulation requires freshly laid eggs [20] our controlled spawning protocol will greatly facilitate experimentation.

It is important to note that our controlled spawning protocol completely eliminates the spontaneous spawning events observed in other systems [9]–[10], [14]–[15]. For the first time, this ensures the controlled production of offspring from specific animals of interest (e.g., after experimental manipulation).

## Concluding Remarks

Our optimized facility provides access to amphioxus adults and embryos throughout the almost entire calendar year, facilitating the establishment and transfer of molecular techniques, the usage of novel imaging techniques and experimental manipulation at much larger scale.

## Supporting Information

Figure S1 Environmental Setup of the Facility. (**A**) Environmental setup in the roof of each main module of the facility, showing: water piping (w), oxygen piping (o) and a complete set of night (ml) and day light (dl) bulbs. (**B**) Faucets for manual control of the water flow in the individual amphioxus boxes (arrow) and the main tanks (double arrow). (**C**) Facility in an open (left) and closed (right) configuration via a black-out automatic blind hosted in the upper blind case (bc) at the top of the facility cabinet. (**D**) Filter unit showing three foam mats of different colours according to the pore diameter (black, blue, red), the biological filter, and on the left side the lower reservoir tank containing the protein skimmer and pump for re-circulation of the purified water.
(TIF)

Figure S2 Water Circuitry of the Amphioxus Facility. Schematic representation of the water circuitry of the amphioxus facility. Only one shelf is represented for clarity. The water is cooled in the upper distribution tank. Before being distributed to the individual amphioxus boxes the water is UV sterilised and pumped under the control of magnetic solenoid valves, which flow can be programmed through the operations panel. Through the netted outlets in the amphioxus boxes, water streams out to the main tanks from where it drains down to the filter unit, the protein skimmer and finally the lower reservoir tank from where the water is pumped back to the upper distribution tank. The facility can be also runt in an open configuration, similar to other systems previously shown (e.g. [2]), if the main tanks are drained by opening the outlet as indicated in the diagram. The air circuitry is also indicated, with pipes supplying oxygen to each of the amphioxus boxes. The inlay is a schematic representation of one of our amphioxus tanks showing: a) netted outlet in the front of the tank; b) feeding vent; c) opening to insert the oxygen tubing; d) opening to insert the water jet.
(TIF)

Figure S3 Operations Panel of the Amphioxus Facility. (**A**) Plan of the inner part of the operations panel cabinet. It contains all electronic connections, fuses and digital controllers of the circuits for the different parts of the amphioxus and algae facility. Al circuits are individualised to avoid a shut down of the entire facility in case of short-circuit in any of the components. Accordingly there are individual electric boxes and fuses for: each of the magnetic valves (for the flow-through system), the two pumps, the UV-light, the daylight bulbs for the amphioxus facility, the daylight for the algae facility, the moonlight leds, the skimmer, the air pump for the amphioxus facility, the air pump for the algae facility, the automatic blind and the pH, salinity and temperature controllers. The digital controllers are equipped with programmable timers to mimic seasonal fluctuations, controlling the flow-through and the sterilisation intervals. Accordingly there are timers for the magnetic valves, for the moonlight and daylight to control the length of the day, the UV lamps for controlling the sterilisation time and another timer for controlling the light in the algae facility (Food Light). (**B**) Front door of the operations panel. It allows changing the mode of operation of the facility into automatic, semi-automatic or manual mode. It includes the buttons to operate the facility manually (pairs of green and red for on and off) and light buttons (only in green) informing of the status of the system. H1–H7 indicates the status of the flow-through per shelf; H8 indicates when it is day in the facility; H9 indicates when it is night in the facility; H4 indicates that the main pump is working (this should be permanently on); H10 indicates when the sterilisation device is in operation. The big red button at the bottom of the panel is to cut the power in case of an emergency.
(TIF)

Figure S4 High-Throughput Algae Culturing Facility. To ensure the fresh production of algae in the large quantities necessary to feed the animals on a daily basis we constructed an algae facility. Our algae facility consists of 8 conical funnels with a capacity of 30 litres (http://www.emsustains.co.uk/fish_hatching_jars.htm). Each funnel is oxygenated through a long air tube, which opens at the bottom of the funnel to continuously agitate the culture and impede the sedimentation of the algae. The funnels are illuminated by daylight bulbs and maintained at a constant temperature of 20°C. The funnels are equipped with outlets to dispense saturated solutions of algae (30.000–80.000 cells/ml) whenever necessary. Our algae facility operates in tandem with our amphioxus facility so with a single operations panel we control and program both facilities. The figure shows the accessory algae facility showing different algal cultures. The 30-litre funnels are mounted in a self-constructed scaffold to allow maximal exposure to the light and to facilitate the manipulation. Daylight bulbs are mounted on the wall and the oxygen system is on the upper part of the facility. All components of the algae facility run under the control of the amphioxus facility through the main operation panel.
(TIF)

Figure S5 Raw Spawning Data. The tables show the total numbers of shocked animals per month, in the course of this investigation. The number of spawning animals in relation to the total shocked was used to calculate the percentage of spawning efficiency per month and year (see Figure 4). Over the time the number of animals unable to spawn after the thermal shock diminished with only occasional individuals registered in 2012.
(TIF)

Materials and Methods S1 Materials and Methods S1
(DOC)

Protocol S1 Amphioxus Care
(DOC)

Protocol S2 Time Lapse Spawning
(DOC)

Table S1 Adult Culturing Setups. The table summarizes the major differences between the prototype and the optimised versions of the amphioxus facility assayed. The graphs compare the annual temperature curves in the sea and in the facility. In all cases same natural topology of temperature curves was reproduced annually, though the range applied in the two setups was different. In the prototype we reproduced exactly the same temperature values as observed in nature, whereas in the optimised facility we reduced the overall annual temperature around 3.5°C in average. SSW: Surface Sea Water; DSW: Deep Sea Water (3 meters deep).
(TIF)

Table S2 Raw Survival Data. The table shows the number of animals collected and the number of survivor over the time in the course of this investigation. Recover after quarantine upon arrival of the animals to the facility is also indicated. Death others: indicate recorded deaths after quarantine but before one year of farming.
(TIF)
