# Supplementary figures and images for: Methods for Generating Year-Round Access to Amphioxus in the Laboratory
Source: PLoS One. 2013 Aug 26;8(8):e71599. doi: 10.1371/journal.pone.0071599 (PMC3753313; doi:10.1371/journal.pone.0071599)

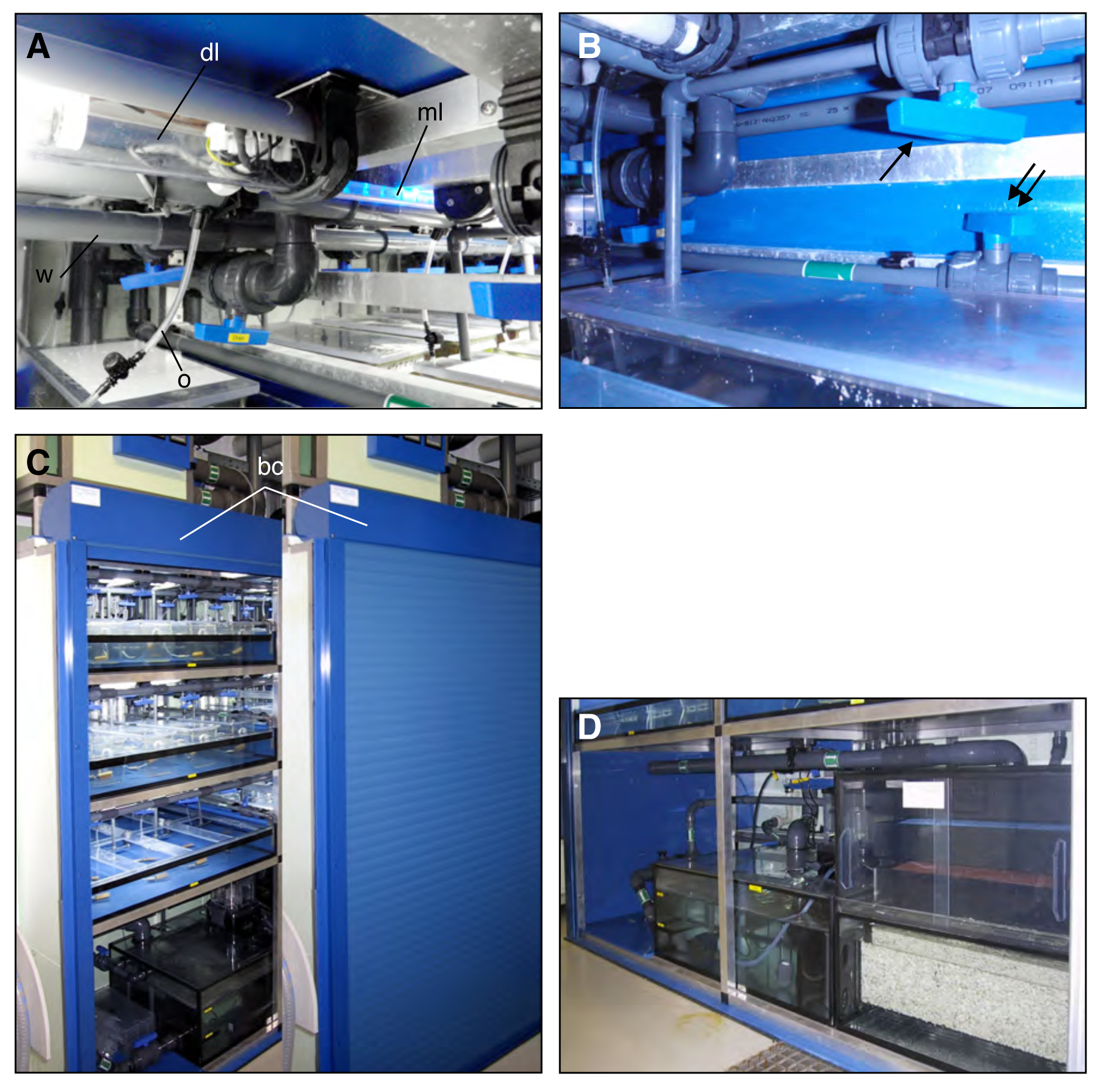

Supplement: Figure S1 — Environmental Setup of the Facility. (A) Environmental setup in the roof of each main module of the facility, showing: water piping (w), oxygen piping (o) and a complete set of night (ml) and day light (dl) bulbs. (B) Faucets for manual control of the water flow in the individual amphioxus boxes (arrow) and the main tanks (double arrow). (C) Facility in an open (left) and closed (right) configuration via a black-out automatic blind hosted in the upper blind case (bc) at the top of the facility cabinet. (D) Filter unit showing three foam mats of different colours according to the pore diameter (black, blue, red), the biological filter, and on the left side the lower reservoir tank containing the protein skimmer and pump for re-circulation of the purified water. (TIF) [file pone.0071599.s001.tif]

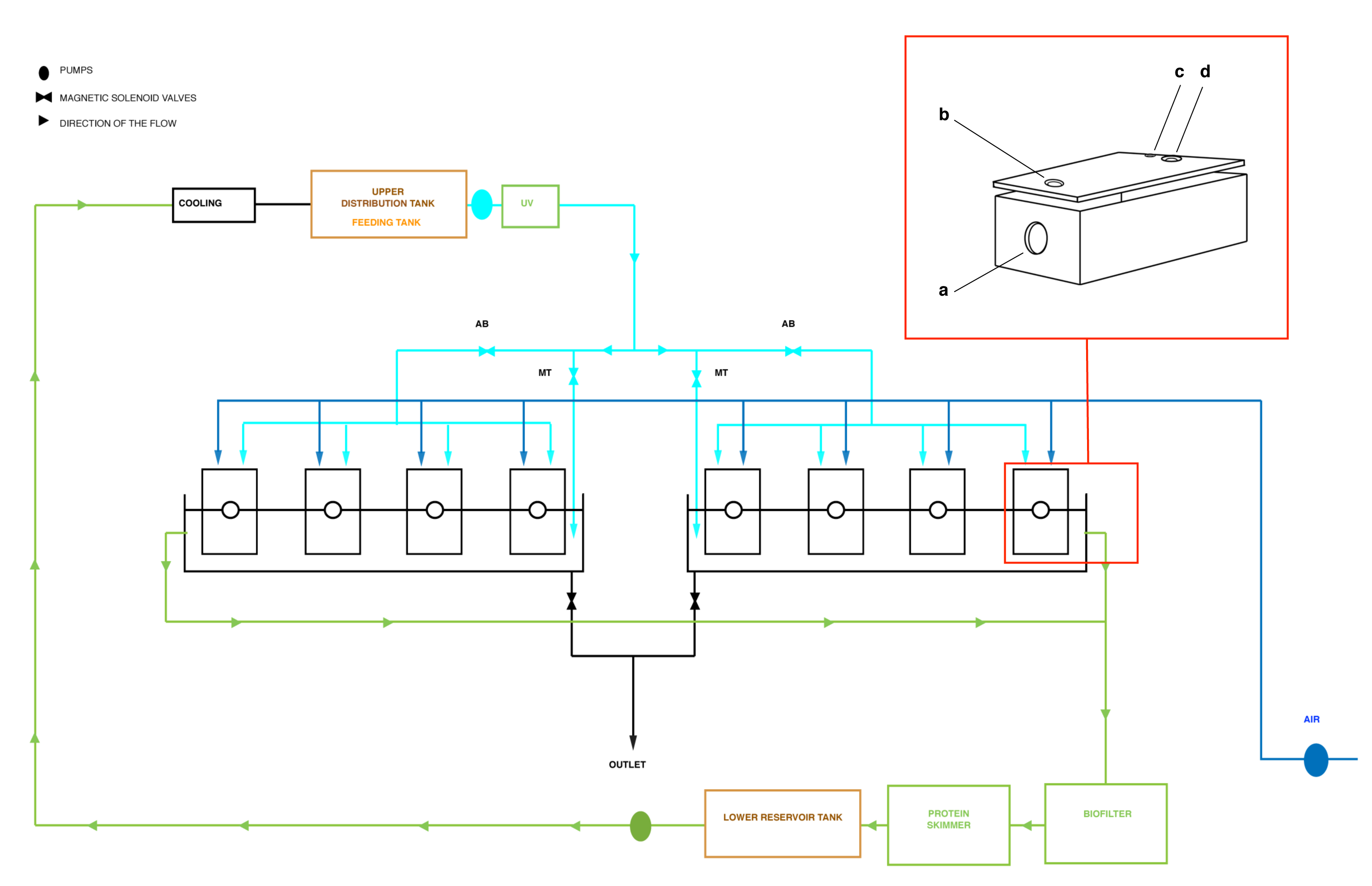

Supplement: Figure S2 — Water Circuitry of the Amphioxus Facility. Schematic representation of the water circuitry of the amphioxus facility. Only one shelf is represented for clarity. The water is cooled in the upper distribution tank. Before being distributed to the individual amphioxus boxes the water is UV sterilised and pumped under the control of magnetic solenoid valves, which flow can be programmed through the operations panel. Through the netted outlets in the amphioxus boxes, water streams out to the main tanks from where it drains down to the filter unit, the protein skimmer and finally the lower reservoir tank from where the water is pumped back to the upper distribution tank. The facility can be also runt in an open configuration, similar to other systems previously shown (e.g. [2]), if the main tanks are drained by opening the outlet as indicated in the diagram. The air circuitry is also indicated, with pipes supplying oxygen to each of the amphioxus boxes. The inlay is a schematic representation of one of our amphioxus tanks showing: a) netted outlet in the front of the tank; b) feeding vent; c) opening to insert the oxygen tubing; d) opening to insert the water jet. (TIF) [file pone.0071599.s002.tif]

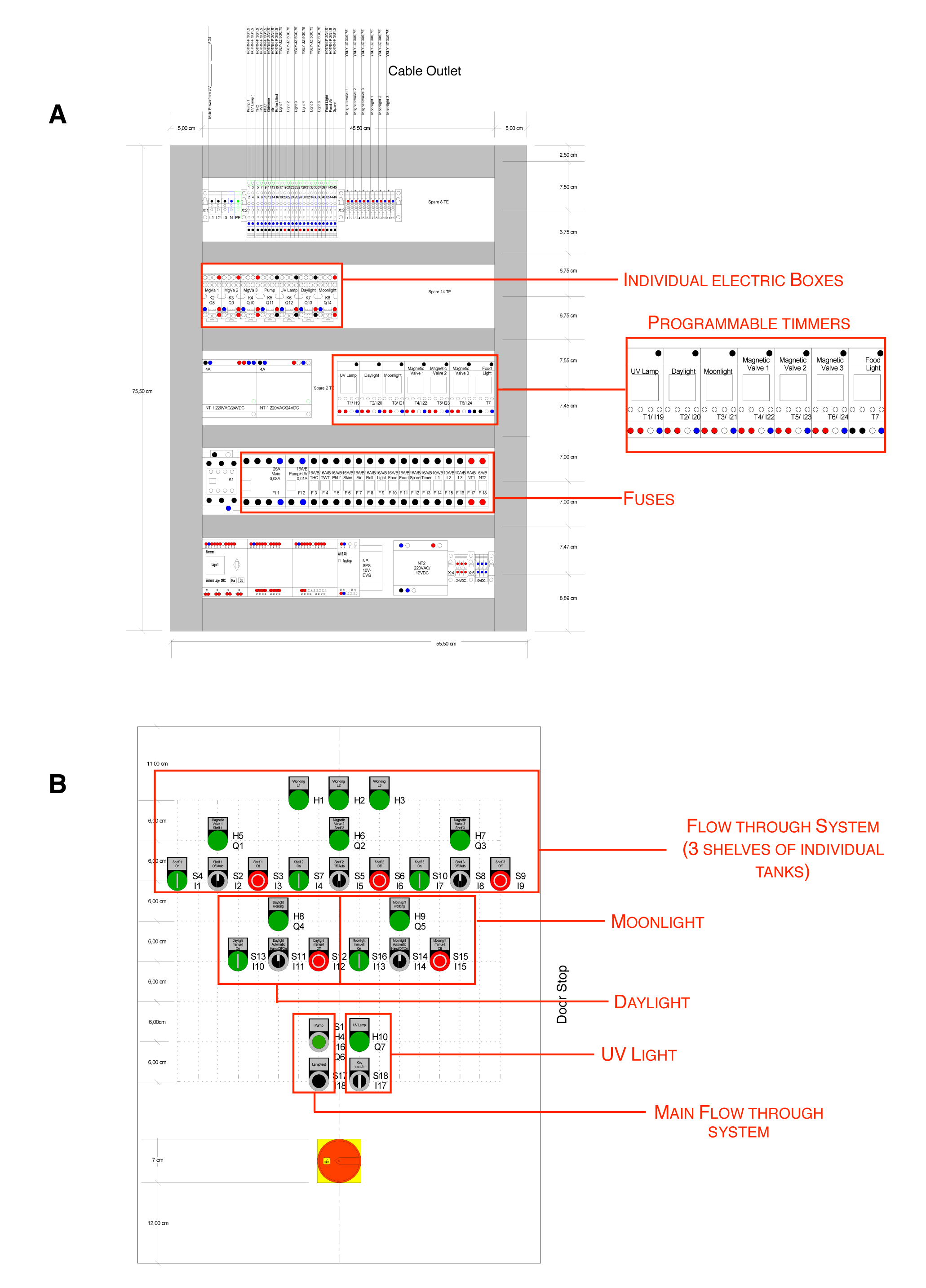

Supplement: Figure S3 — Operations Panel of the Amphioxus Facility. (A) Plan of the inner part of the operations panel cabinet. It contains all electronic connections, fuses and digital controllers of the circuits for the different parts of the amphioxus and algae facility. Al circuits are individualised to avoid a shut down of the entire facility in case of short-circuit in any of the components. Accordingly there are individual electric boxes and fuses for: each of the magnetic valves (for the flow-through system), the two pumps, the UV-light, the daylight bulbs for the amphioxus facility, the daylight for the algae facility, the moonlight leds, the skimmer, the air pump for the amphioxus facility, the air pump for the algae facility, the automatic blind and the pH, salinity and temperature controllers. The digital controllers are equipped with programmable timers to mimic seasonal fluctuations, controlling the flow-through and the sterilisation intervals. Accordingly there are timers for the magnetic valves, for the moonlight and daylight to control the length of the day, the UV lamps for controlling the sterilisation time and another timer for controlling the light in the algae facility (Food Light). (B) Front door of the operations panel. It allows changing the mode of operation of the facility into automatic, semi-automatic or manual mode. It includes the buttons to operate the facility manually (pairs of green and red for on and off) and light buttons (only in green) informing of the status of the system. H1–H7 indicates the status of the flow-through per shelf; H8 indicates when it is day in the facility; H9 indicates when it is night in the facility; H4 indicates that the main pump is working (this should be permanently on); H10 indicates when the sterilisation device is in operation. The big red button at the bottom of the panel is to cut the power in case of an emergency. (TIF) [file pone.0071599.s003.tif]

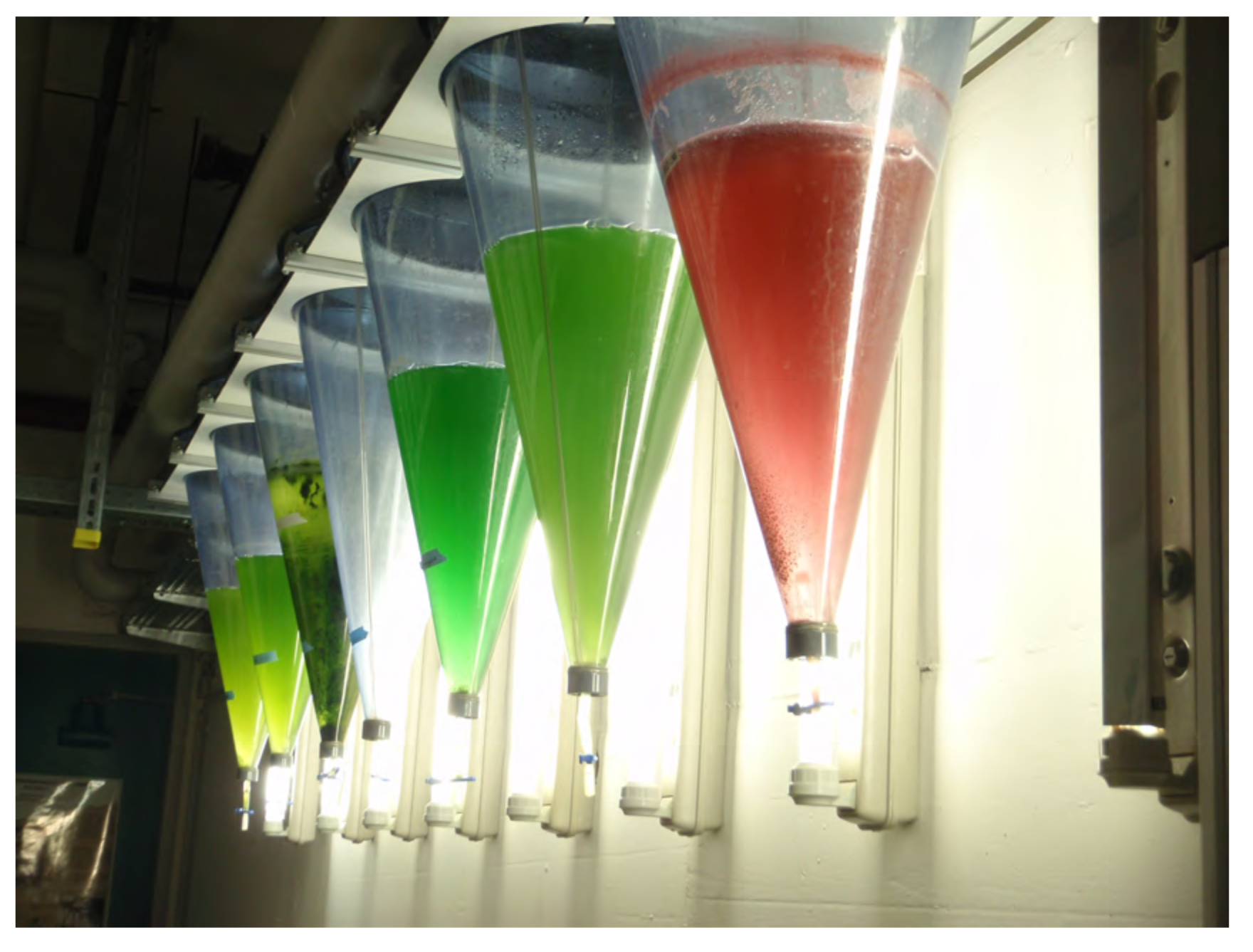

Supplement: Figure S4 — High-Throughput Algae Culturing Facility. To ensure the fresh production of algae in the large quantities necessary to feed the animals on a daily basis we constructed an algae facility. Our algae facility consists of 8 conical funnels with a capacity of 30 litres (http://www.emsustains.co.uk/fish_hatching_jars.htm). Each funnel is oxygenated through a long air tube, which opens at the bottom of the funnel to continuously agitate the culture and impede the sedimentation of the algae. The funnels are illuminated by daylight bulbs and maintained at a constant temperature of 20°C. The funnels are equipped with outlets to dispense saturated solutions of algae (30.000–80.000 cells/ml) whenever necessary. Our algae facility operates in tandem with our amphioxus facility so with a single operations panel we control and program both facilities. The figure shows the accessory algae facility showing different algal cultures. The 30-litre funnels are mounted in a self-constructed scaffold to allow maximal exposure to the light and to facilitate the manipulation. Daylight bulbs are mounted on the wall and the oxygen system is on the upper part of the facility. All components of the algae facility run under the control of the amphioxus facility through the main operation panel. (TIF) [file pone.0071599.s004.tif]

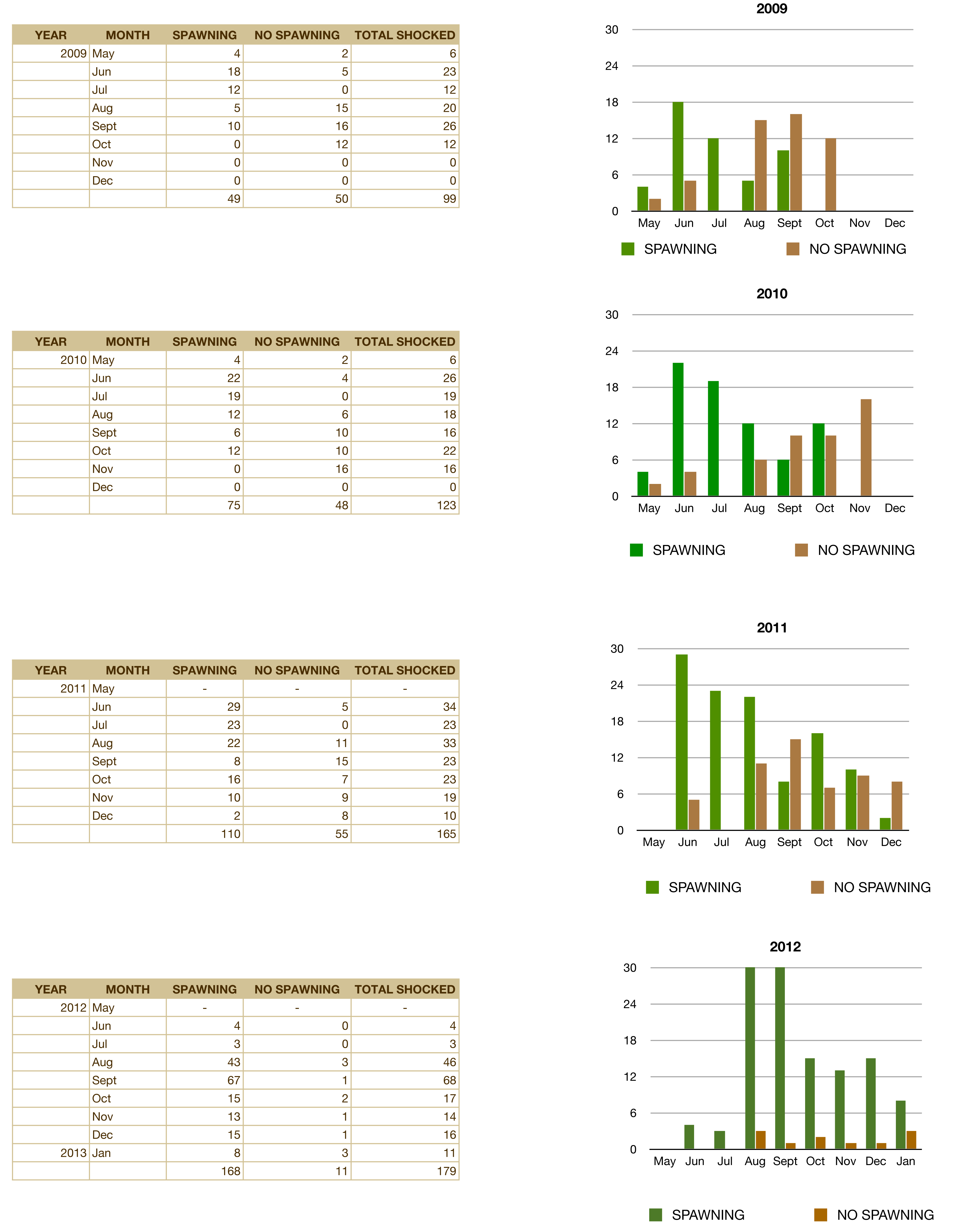

Supplement: Figure S5 — Raw Spawning Data. The tables show the total numbers of shocked animals per month, in the course of this investigation. The number of spawning animals in relation to the total shocked was used to calculate the percentage of spawning efficiency per month and year (see Figure 4). Over the time the number of animals unable to spawn after the thermal shock diminished with only occasional individuals registered in 2012. (TIF) [file pone.0071599.s005.tif]

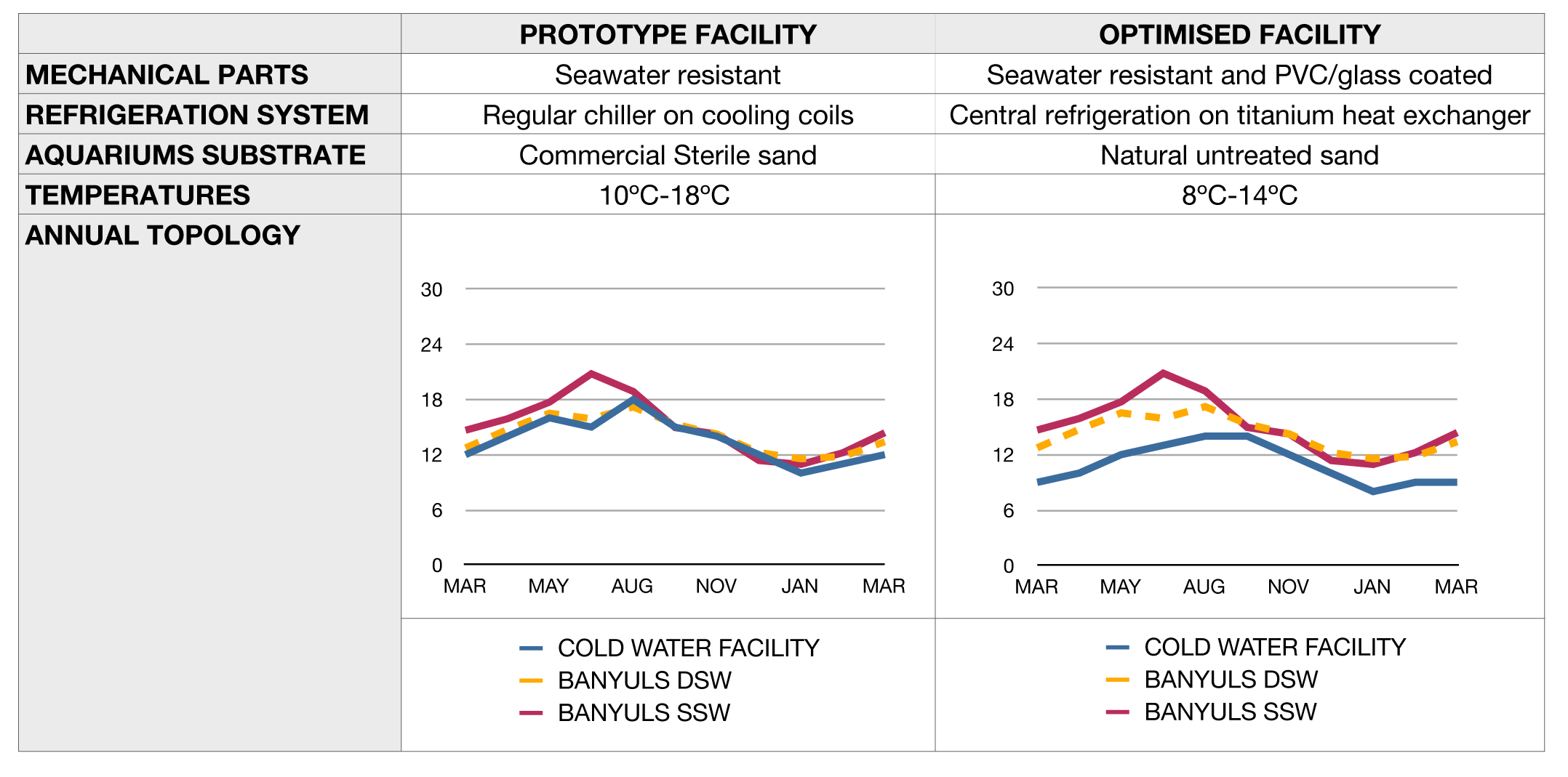

Supplement: Table S1 — Adult Culturing Setups. The table summarizes the major differences between the prototype and the optimised versions of the amphioxus facility assayed. The graphs compare the annual temperature curves in the sea and in the facility. In all cases same natural topology of temperature curves was reproduced annually, though the range applied in the two setups was different. In the prototype we reproduced exactly the same temperature values as observed in nature, whereas in the optimised facility we reduced the overall annual temperature around 3.5°C in average. SSW: Surface Sea Water; DSW: Deep Sea Water (3 meters deep). (TIF) [file pone.0071599.s009.tif]

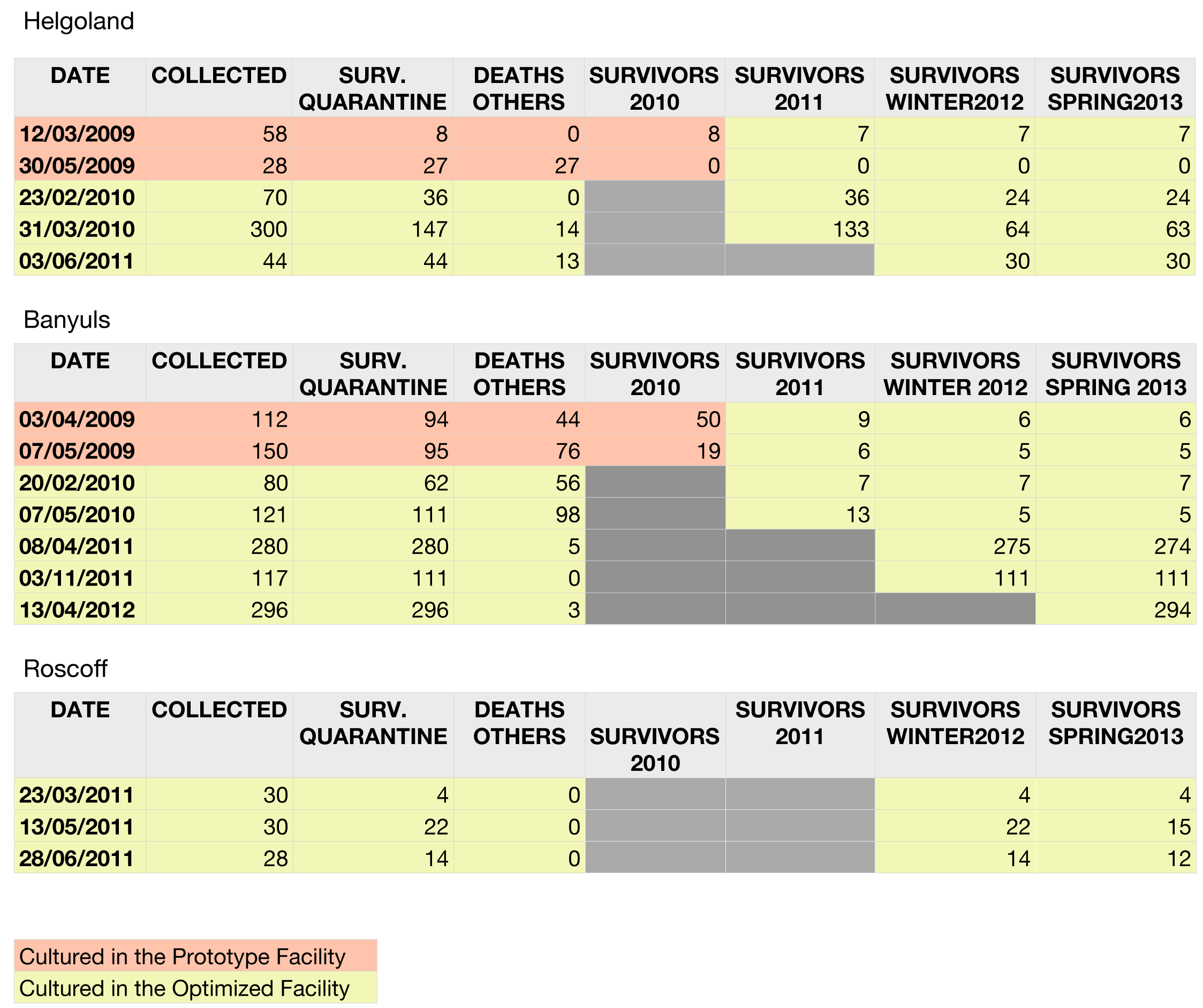

Supplement: Table S2 — Raw Survival Data. The table shows the number of animals collected and the number of survivor over the time in the course of this investigation. Recover after quarantine upon arrival of the animals to the facility is also indicated. Death others: indicate recorded deaths after quarantine but before one year of farming. (TIF) [file pone.0071599.s010.tif]
