# Supplementary material for: Methods for Generating Year-Round Access to Amphioxus in the Laboratory
Source: PLoS One. 2013 Aug 26;8(8):e71599. doi: 10.1371/journal.pone.0071599 (PMC3753313; doi:10.1371/journal.pone.0071599)
Supplement: Materials and Methods S1 — (DOC) [file pone.0071599.s006.doc]

**MATERIALS AND METHODS S1**

Design and rationale of the amphioxus facility

In order to simulate as closely possible the benthic marine ecosystem where wild amphioxus live, we engineered an automated marine facility running on constantly flowing-through natural sea water, with natural daylight, dusk and dawn, natural moon phases and natural sand-bedding (Fig. 1). The facility is designed and built to operate continuously, with the aim of hosting amphioxus permanently and therefore generating laboratory-based amphioxus colonies. Although it can be run in an open configuration, the facility has been conceived as a self-sustained platform, meaning that no incoming seawater is needed for the daily maintenance of the system. This is possible due to our newly designed encapsulated tank system and the thermal insulation of the entire facility. Both features together contribute to minimize seawater evaporation, thereby producing invariable and finely controlled values of salinity and pH. This way, the closed seawater circuit has the advantage of maintaining the properties of the water unchanged and devoid of any influence from incoming external seawater. Furthermore, all parameters are controlled through electronic sensors and high standards of water quality are achieved through the self-purifying water circulation system, which recycles the seawater through a series of mechanical and biological filters, a protein skimmer and a UV sterilisation device. Additionally, the facility is lightproof, enabling the light cycle to vary as in nature, albeit timed to experimental requirements. Programmable light/temperature fluctuations prove here as an essential tool to be able to manipulate the time and duration of the breeding periods in the laboratory, which is essential for experimentation *in vivo*. The facility also incorporates major technical developments, for the first time implemented into an aquarium system. These are a powerful refrigeration system that can generate temperatures as low as 7ºC, PVC coated mechanical components and a comprehensive control panel to finely control all parameters inside the facility. Such technical features were developed to achieve high survival rates on a controlled seasonally fluctuating environment, intended to mimic the natural conditions. The rational behind is to produce a balanced and antibiotic-free environment that ensures the welfare of permanent amphioxus colonies in the laboratory, not only in terms of survival but also in terms of completing their reproductive cycles as in nature.

Encapsulated tank system

The basic frame of the facility is based on a standard Xenopus facility. As opposed to a zebrafish facility the tanks are equally integrated in the structure but they are broader (Fig. 1A). This was the design of choice for the main tanks, in which the actual amphioxus aquariums are bathed. The facility is divided into six of these main tanks distributed in three different shelves. Every main tank encapsulates eight husbandry boxes for amphioxus distributed in two rows of four (Fig. 1B). The amphioxus boxes are held in the main tank by a fitted PVC grid, leaving two thirds of their height immersed in the main tank (Fig. 1C). The grid has a dual function, while supporting the amphioxus boxes it also seals the entire main tank for minimizing water evaporation and air-temperature exchange. The encapsulated tank system in addition ensures homogeneous conditions in all tanks within a module. This is achieved by a continuous flow of water in the main tanks. Because both the husbandry boxes and the main tanks are integrated in the same seawater circuit it is possible to flush the amphioxus boxes without temperature or salinity fluctuations. Without breaking the seal, seawater is pumped into the individual amphioxus boxes through water jets inserted in PVC lids. These PVC lids cover the amphioxus boxes individually and are also custom-perforated to host the oxygen tubes. This facilitates the manipulation of individual amphioxus tanks without perturbing the rest of the colony.

Environmental Setup

Every main module has an independent built-in tubing system for oxygen and water, and a complete set of night and day light bulbs (Fig. S1A). The oxygen is insufflated by a high-pressure air pump to a maximum speed of 1800 l/h. Small knobs in the oxygen tubes allow to regulate manually the oxygen flux to the desired strength (Fig. S1A). The oxygen is constantly supplied to each tank by dispersion through 5 cm air stones (Fig. 1D). Water is also distributed by dispersion through multi-perforated water jets. The inverted T-shape of the water jets, multi-perforated at the base (Fig. 1D), was conceived to generate a unidirectional seawater wave as wide as the width of the amphioxus boxes. This ensures the entire surface of the tanks to be washed out and prevents internal differences of the flow strength within the tank. The water flow is controlled at two levels operating simultaneously: manually through the faucets at the junction of the water jet inlet (Fig. S1B) and, automatically or manually through magnetic solenoid valves located at the entry of the main upper pipe in each main module.

Natural lighting is provided through daylight fluorescents, the intensity of which is adjustable, changing the colour as in nature, therefore enabling a dim phase to mimic dusk and dawn. The fluorescents gradually increase their intensity from 0% to 100% in 40 minutes at dawn and, vice versa at dusk. The dimming phase is in turn accompanied by rise or decrease of the moonlight, which is mimicked through blue led bulbs emitting on wavelengths of 465-475 nm. Following the same principle as the daylight, the moonlight gradually increases the intensity during twilight but the maximum percentage of intensity is only that representing the particular moon phase for each given day, for example, from 0% to 50% to simulate a half moon night. Since amphioxus are extremely sensitive to light exposure, the facility is light-proof and equipped with an automatic roller-blade blind (Fig. S1C), to ensure that light intensities and cycles are strictly produced as programmed.

The control of the temperature in the facility is another of the new features implemented in our system. Not only allows an ample range of temperatures but also the lowest ever incorporated in an aquarium system. Via coupling to the refrigeration system of the building, the facility is able to sustain temperatures as low as 7ºC. This is finely controlled by a heat exchanger that cools down the pumped water as it enters in the distribution tank, also thermally isolated to prevent air-temperature exchange.

Seawater circuitry

The facility is running in a closed circuit of 700 litres of natural seawater. The natural sea water is provided by the Biologische Anstalt in Helgoland- Bremerhaven from close to the natural amphioxus habitat and transported by Hanke in 300 litres containers. This water is mixed with smaller amount of seawater hand-collected in Banyuls-sur-mer to achieve salinity of 54-56ms and a consensus pH of 7.9-8.1. In the upper distribution tank, the water is cooled down and UV sterilized before being distributed to the individual amphioxus boxes and the main tanks (Fig. S2). From the distribution tank water is pumped to a maximum speed of 2200 litres per hour and it is dispensed under the control of magnetic solenoid valves. Different sets of solenoid valves are located either at the main upper pipe in each module, for the amphioxus boxes, or in the lateral pipes in each module, for the main tanks (Fig. S2). In the main tanks, as described above for the amphioxus boxes, faucets operating in parallel with the solenoid valves are also regulating the final water flow. In the case of the main tanks, faucets for a manual control of the water flow are located just above the PVC grid of the encapsulated system (Fig. S1B). In the course of this investigation, continuous water flow was provided to the main tanks by setting the valves in a constant open configuration. Conversely, the valves supplying the amphioxus boxes were finely programmed to generate a continuous flow-through at intervals to allow the animals to feed. All amphioxus boxes are equipped with netted outlets to allow the flow-through propelled by the water jets (Fig 1C). Therefore, apart from their role as temperature homogenizers, the main tanks also act as a water collection system. From the main tanks, water flows via gravitation to the filter unit, constructed at the base of the facility (Fig. S1D). Once in the filter unit, the water is cleaned through a series of mechanical and biological filters and finally, in the lower reservoir tank, protein skimmed. From the lower reservoir tank the water is pumped up back to the upper distribution tank, starting the cycle again (Fig. S2).

Control Panel

The operations panel is the electronic circuit controlling all parameters in the facility (Fig. S3). This includes: a) mechanical parts: pumps, blinds and magnetic valves; b) physical parameters of the water: temperature, oxygen, salinity, pH, protein skimmer and other systems of water purification (e.g. UV light); c) environmental conditions: seasonal fluctuations of temperature, day and night cycles and moon phases. Apart from that, it also controls our accessory algae facility, also subjected to programmable control of daylight cycles and oxygen pumping (Fig. S4).

The control panel allows the facility to be runt in automatic, semi-automatic or manual mode, depending on the circumstances and/or experimental needs. In the automatic mode, seasons can be programmed for the entire year with appropriated changes in day-night ratios and temperature. In the course of this pilot investigation it was runt in a semiautomatic mode. This allowed us to reproduce wild conditions for newly collected animals upon arrival, to facilitate their gradual adaptation to the system. As for the experimental optimization of the facility, the semiautomatic mode permitted us to modify different parameters according to the response of the animals.

Technical data

The facility is built in a lightproof cabinet with the following dimensions: 195,60 X 222,80 X 112,5 cm (Fig. 1A). At the front, the facility closes with an automatic blind whenever accessing the animals is unnecessary. The interior of the cabinet hosts our newly designed encapsulated tank system distributed in three shelves and consisting of: two main glass tanks each shelf (92 x 95 x 13 cm), encapsulating eight amphioxus boxes each main tank. The amphioxus boxes are made of polycarbonate, with a practicable capacity of 5 litres (34 x 15 x 16,5 cm), with adjusted individual PVC lids (34 x 15 cm) and inserted into a custom perforated PVC grid (8 rectangles of 34 x 15 cm in a panel of 92 x 95 cm), which seals the entire surface of each main tank (Fig. 1B-C). This minimizes evaporation in the facility’s closed water circuitry, which consists of 700 litres of constantly flowing natural seawater powered by two seawater-proof pump systems. The first pump system is located in the upper distribution tank with a performance of 2200 litres per hour and equipped with cooling chambers (Quiet One 2200, Lifegard Aquatics). The second pump system, located in the lower reservoir tank, is a powerful self-priming centrifugal pump able to mobilize up to 7000 litres of water per hour (NK-25B, Kripsol). The latter pumps the water into the upper distribution tank, just after the water is purified. The water purification occurs next to the lower reservoir tank where the filter unit, composed of mechanical filters (foam mats 10, 20 and 30 PPI; Aqua Schwarz), biological filters (Coral sand granulation =15, Aqua Schwarz; Clear-Flo Bacterial starter and Biobooster, Söll; Baktoplan Marin, Preis Aquaristik) and a protein skimmer (C-Skim 1200; Red Sea), is built-in. The water purification circuit starts when water coming from the amphioxus boxes is collected in the main tanks, from where it drains down into the filter unit. Once there, the water percolates through the serially arranged foam mats, the denitrifying bacteria in the biological filter and the protein-skimmer, which removes any remaining organic compounds still in the water. Just before being re-distributed, the water is UV sterilized (P-R177300 QL-40, Lifegard Aquatics) at the top of the facility cabinet (Fig. 1A). The re-distribution of the water into the amphioxus boxes and the main tanks is controlled via magnetic solenoid valves (SIBO solenoid valve PVC). These can operate either automatically through the timer installed in the facility (see Fig. S3) or manually, so the animals can be fed at intervals when the flow-through is inactivated.

Since the amphioxus facility was designed to mimic natural conditions in the wild, we use daylight fluorescents that change colours according to time of day including dusk and dawn (Lumilux Daylight 860, OSRAM) and blue LED bulbs (Moonlight bar with 12 LED, Aqua Schwarz GmbH) emitting wavelengths of 465-475nm to mimic moonlight (Fig. 1E and 1F). Twilight time was programmed to last for 40 minutes for the European amphioxus. The animals were maintained in a semi-inverted light cycle, with the night starting at 12:00 (noon), to facilitate maintenance and experimental work on a daily basis. Day:night ratios are as follows: 14:10 for mimicking summer, 10:14 for mimicking winter, and gradually inverted from summer to winter, and vice versa, to mimic spring and autumn conditions, always in tandem with respective changes in temperature.

Oxygen was constantly provided by dispersion through 5 cm air stones (Fig. 1D), via a high-pressure air pump to a maximum speed of 1800 litres per hour (Silenta Pro 1800, Velda). Seawater was continuously refreshed through propulsion by water jets installed at the rear of each box. Water jets, multi-perforated at the base, generate unidirectional flow along the entire width of the boxes (Fig. 1D), washing out the entire surface of the aquariums through netted outlets located at the front (Fig. 1C).

For temperature, pH and salinity control, the facility is equipped with highly sensitive digital controllers (Temperature: T-2001-CC; pH: PH-2001-C, Aqua Medic; Salinity: LF-2001-C, Aqua Medic), which send information every fifteen minutes to a central computer. Sensors are installed in the upper distribution tank from where water reaches individual boxes and main tanks.

Further information on all components can be obtained from Aqua Schwarz GmbH, who assembled and built our facility at EMBL and provided most of the parts (Aqua Schwarz GmbH,  Maschmühlenweg 40-42,  D-37081 Göttingen,  Germany).

Culturing conditions

Prototype Facility: The initial design was equipped with a regular seawater chilling system based on internal cooling coils, generating minimal temperatures of 10ºC-12ºC. Although, temperatures were a little unstable in the lower range, the setup still well-approximated natural conditions (Table S1). The annual range of implemented temperatures was 10ºC-18ºC. All parts of the facility achieved high standards of seawater resistance and stainless steel was used to construct pH, salinity and temperature control units. All boxes contained commercial sterile sand. Since amphioxus show granulometry preferences [1], tanks were bedded with commercial sand ranging from 2 to 0.1mm in diameter (Preis Alpha-Marin and Bora-Bora; Preis-Aquaristik). Different grain sizes were tested independently and in combination.

Optimised Facility: A central cooling system was connected via a titanium heat exchanger (Type D-TWT-35, Aqua Schwarz GmbH), enabling stable temperatures down to 7ºC. The annual temperature range was 8ºC-14ºC (Table S1). High precision in temperature, pH and salinity control was achieved through digital controllers. Respective control units were custom-covered in glass or made of graphite (the salinity controller LF-2001-C). Likewise, all mechanical parts, apart from achieving high standards for seawater resistance, were further coated in PVC as an extra safety measure against corrosion, protecting the facility against a wider spectrum of seawater types. All boxes contained natural sand, brought from the site of collection. Unlike previous protocols [2] the natural sand was left untreated to keep the meio-benthos and microbial communities intact and as close as possible to their natural state.

Diet and supplements

Since the natural amphioxus diet is largely unknown, amphioxus are commonly fed on a mix of three or four microalgae [2-6]. Our animals are fed on seven algal species grown in a custom-made algae facility (see Fig. S4). As starter cultures, we used *Tetraselmis* sp. (SAG Number: 3-98); *Spirulina labrynthiformis* (SAG Number: 59-90); *Dunaliella tetriolecta* (SAG Number: 13-86); *Isochrysis* sp. (SAG Number: 927-2); *Porphyridium purpureum* (SAG Number: 1380-1d); *Monochrysis sp*. (CCAP Number: 931/2); *Nanochloropsis salina* (Interaquaristik Number: 01176). Starter cultures were used to inoculate 2 litres of seawater enriched with Guillard’s (F/2) solution (SIGMA: G0154). One of litre of this preparation is then used to inoculate the 30-litres-funnels, initially containing 5 litres of freshly prepared Guillard’s solution 1x, in seawater. When the algae become visible in the funnels, these are topped up to 30 litres with fresh Guillard’s solution 1x in seawater. Around 8 litres of fresh algal suspension are needed per day to maintain the culture. Different combinations of algae were alternated on a daily basis and dispensed at concentrations around 30.000-80.000 cells/ml.

As our algal suspension does not supply other nutrients, such as those contained in zooplankton, we tested different food mixes with or without supplements:

-FOOD MIX A (for adults): Fresh algae mix consisting of 3 volumes of green algae per 1 volume of red/brown algae.

-FOOD MIX B (for adults): Food Mix A with the following added supplements: Planktomarine (GroTech), Nutrimarin (Grotech), Vitamino (Grotech).

-FOOD MIX C (for adults): Food Mix A with the following added supplements: Planktomarine, Nutrimarin, Vitamino, Liquifry (Interpret), Rotirich ([http://www.aquaticeco.com](http://www.aquaticeco.com/)) and iodine (JBL).

-FOOD MIX D (for embryos): Fresh algae mix consisting of 2 volumes of brown algae per 1 volume of green algae.

-FOOD MIX E (for embryos): Food Mix D with the following added supplements: Food Mix A with the following added supplements: Planktomarine, Nutrimarin, Vitamino, Liquifry and iodine.

In all cases, supplements were added in the concentrations recommended by the manufacturer. All food was filtered before dispensing through a 200 micron mesh for adults, or a 5 micron mesh for embryos.

Aproximated Building and Running Costs

- Optimized Facility in the configuration and dimensions here presented: 70.000 €
- Annual Facility Maintenance Contract with Aqua Schwarz: 500€ (per year)
- Natural water collection and transportation: 1480 € (per year)
- Algae Facility: 800 €
- Food Supplements: Around 300€ (per month)

**SUPPORTING INFORMATION REFERENCES**

1. Desdevises Y, Maillet V, Fuentes M, & Escriva H. (2011). A Snapshot of the Population Structure of Branchiostoma lanceolatum in the Racou Beach, France, during Its Spawning Season. *PLoS ONE* **6**:e18520.
2. Theodosiou *et al*. (2011) Amphioxus spawning behavior in an artificial seawater facility. *J. Exp. Zool. Part B: Molec. and Dev. Evol.* **316**: 263-275.
3. Fuentes *et al*. (2004) Preliminary observations on the spawning conditions of the European amphioxus (Branchiostoma lanceolatum) in captivity. *J. Exp. Zool. Part B: Molec. and Dev. Evol.* **302**: 384–391.
4. Fuentes *et al*. (2007) Insights into spawning behavior and development of the european amphioxus (Branchiostoma lanceolatum)*. J. Exp. Zool. Part B: Molec. and Dev. Evol.* **308**: 484-493.
5. Yasui K, Urata M, Yamaguchi N, Ueda H, Henmi Y (2007) Laboratory Culture of the Oriental Lancelet Branchiostoma belcheri. *Zoological Science* **24**: 514–520.
6. Holland LZ, Yu JK (2004) Cephalochordate (amphioxus) embryos: procurement, culture, and basic methods. *Methods Cell Biol* **74:**195-215.
