# Supplementary material for: Methods for Generating Year-Round Access to Amphioxus in the Laboratory
Source: PLoS One. 2013 Aug 26;8(8):e71599. doi: 10.1371/journal.pone.0071599 (PMC3753313; doi:10.1371/journal.pone.0071599)
Supplement: Protocol S1 — Amphioxus Care (DOC) [file pone.0071599.s007.doc]

**PROTOCOL S1: AMPHIOXUS CARE**

Animal transportation and adaptation to the facility

In all cases the animals were transported in plastic bags containing 5 cm of natural sand, seawater and pressurized oxygen. The transportation was performed in thermostable containers to keep the animals cool.

During the transportation the animals were kept in the dark. Congruently, all animals were introduced to the facility during the night to avoid light shocks.

Adaptation of the collected batches was done gradually:

-DAY 1: Introduction of transport plastic bags into the facility. The sole objective is to gradually equilibrate the temperature of the transported seawater. Fresh air from the facility is supplied to the open bags to avoid anoxia after the trip.

DAY 2: Bag contents are released into a fresh tank. While the animals are inside the sand, the water of origin is filtered through a 200 micron-mesh. The objective is to eliminate the faeces deposited by the animals during the transportation and other solid debris without disturbing the natural meio-benthos in the natural sand.

DAY 3-6: Replacement of the seawater of origin. Only one quarter of the total volume of the tank is replaced daily. After four days the entire tank should be filled with seawater from the facility, so the tank can enter in circulation by opening the outlet. From the third day onwards the animals can be fed.

After the gradual adaptation of the freshly collected batches the animals can be redistributed with a density of around 25 animals per tank.

Quarantine

After gradual adaptation of the wild animals to the system, the health of the animals is assessed by behaviour (animals outside the sand or inability to respond to light stimuli) or by examination under the stereoscope (skin lesions, pigmentation or depigmentation, muscle cysts or other kind of parasites usually seen in the atrial cavity). If any pathological symptoms are observed the treatment is customized by supplementing the sea water as follows:

-For mild bacterial infection: Unimarin, following the manufacturer’s instructions (Aquarium Münster Pahlsmeier GmbH, Galgheide 8, D-48291 Telgte, Germany)

-For strong bacterial infection: Penicillin/Streptomicin (10ml/l) (SIGMA: P0781)

-For parasite infestation: Gyromarin, following the manufacturer’s instructions (Aquarium Münster Pahlsmeier GmbH, Galgheide 8, D-48291 Telgte, Germany)

- Unknown origin of the disease: Ektomarin, following the manufacturer’s instructions (Aquarium Münster Pahlsmeier GmbH, Galgheide 8, D-48291 Telgte, Germany)

In all cases the quarantine tank is isolated from the rest of the facility by blocking the outlet of the tank. Once the animals are back in the sand and show no symptoms of disease the tank can go back into circulation after four days of unsupplemented water change. Thorough elimination of any traces of antibiotics/drugs is extremely important to avoid damaging the bacteria in the biological filters and other microbia belonging to the natural substrate.

For long-term maintenance all tanks are surveyed daily. If any of the symptoms described arise at any time after adaptation, the animals should be transferred into quarantine tanks as described above.

Long-term maintenance

All tanks are replaced and sand is washed every three weeks.

1. Prepare a fresh large tank to filter the animals out. Water is always from the facility to avoid temperature or salinity shocks.
2. Filter the animals out by sieving the content of the tanks.
3. Collect the animals in the sieve and transfer them to a new tank.
4. Wash the sand that has been separated from the animals with seawater from the facility.
5. When the seawater used for washing the sand becomes clear place it back in the new tank containing the animals.

The protein skimmer and filters are cleaned once a week and the water pumps once every six months, following the manufacturers instructions in each case.

Feeding

It is widely known that amphioxus filter feed on plankton and other small particles, yet the composition of the uptake in the wild is unknown. In our facility the animals are fed daily with a mix of freshly grown algae. This is intended to recreate the diversity of the phytoplankton source but does not provide other nutrients that the animals in the wild probably uptake from zooplankton. Bearing this in mind, we investigated the utility of a number of commercially available supplements. These included essential amino acids, vitamins, iodine and fatty acids. Below we describe the formula that allowed us to boost our survival rates in the facility and that replenished the gonads of our animals inside and outside the natural spawning season (Fig. 5).

1. Mix per tank:

-150 ml of freshly collected algae (3:1, green algae: red algae)

-2.5 ml of Rotirich ([http://www.aquaticeco.com](http://www.aquaticeco.com/))

-2.5 ml of Planktomarin (GroTech)

-0.5 ml of Liquifry (Interpret)

-0.5 ml of Nutrimarin (GroTech)

1. Use a blender for homogenising the mix and triturate macroalgae (e.g. spirulina)
2. Filter the mix through a 200 microns mesh.
3. Dispense: Food can be dispensed in two different ways:
4. By dispensing the food mix to each individual amphioxus tank. This can be done, as here, with a squeeze bottle with sprout through the feeding whole in each of the lids in each amphioxus tank (See inlay in Fig. S2).
5. By adding the food mix directly into the upper distribution tank / feeding tank (Fig. S2). This second option requires turning off the UV lamp at the time the food is being dispensed.
