# Supplementary material for: Methods for Generating Year-Round Access to Amphioxus in the Laboratory
Source: PLoS One. 2013 Aug 26;8(8):e71599. doi: 10.1371/journal.pone.0071599 (PMC3753313; doi:10.1371/journal.pone.0071599)
Supplement: Protocol S2 — Time Lapse Spawning (DOC) [file pone.0071599.s008.doc]

**PROTOCOL S2: TIME LAPSED SPAWNING**

Spawnings were induced as described in [4] with modifications.

DAY 1 IN THE MORNING

1. Select animals with mature gonads under the scope
2. Transfer the mature animals to cups filled with seawater from the facility (at 14ºC): one cup for males and one cup for females.
3. Transfer the cups to a water bath at 21ºC

No oxygen or food is provided to the animals

DAY 2 IN THE MORNING

1. Collect the water in the cups (already established at 21ºC)
2. Filter the collected water through 0.2 micron mesh and distribute it in new cups. 1cm of filtered water per cup.
3. Transfer one individual per cup
4. Cups are placed in the light box (at 21ºC)
5. Program the light to turn off as desired to obtain sequential spawnings according to the experimental needs.

Minimum time at 21ºC for effective spawning is 36h (including day1), maximum time 46h.

DAY2 IN THE AFTERNOON

1. Collect the eggs and the sperm respectively
2. Transfer the adults to a tank with fresh seawater at 21ºC
3. Transfer the tank to the facility to allow a gradual cooling of the water.

Cryopreservation of amphioxus sperm

Our freezing formula for sperm cryopreservation significantly differs from others previously reported [2]. In our hands, egg yolk in the cryopreservation solution prevents the embryos from hatching at the early neurula stages. These differences might be due to longer preservation times in our case, since we used sperm that had been frozen for years. The facility provides both sperm and eggs routinely but in case of stallions, best results for sperm preservations were obtained following the protocol below:

1. Collect fresh sperm on ice in a 15 ml falcon tube
2. Let the sperm sit down and remove debris from the bottom of the tube to clean the sperm
3. Centrifuge 5 ml of clean sperm in fresh seawater 3000 rpm, 15 minutes at 4ºC
4. Discard supernatant
5. Resuspend the sperm pellet in 1ml of cold fresh seawater by gently pipetting
6. Transfer the 1 ml of resuspended sperm into a 2ml cryotube
7. Add 1ml of and stock of 40%DMSO in cold fresh seawater (final concentration of DMSO = 20%)
8. Seal the tube and freeze it with liquid N_2_ vapours (never put the cryotube in direct contact with the liquid N_2_)
9. When sperm is frozen transfer to a liquid N_2_ long storage container (e.g. cell lines preservation back)
